# Comparison of the effects of oxidative and inflammatory stresses on rat chondrocyte senescence

**DOI:** 10.1038/s41598-023-34825-1

**Published:** 2023-05-11

**Authors:** Misaki Yagi, Kentaro Endo, Keiichiro Komori, Ichiro Sekiya

**Affiliations:** Center for Stem Cell and Regenerative Medicine, Tokyo Medical and Dental University (TMDU), 1-5-45 Yushima, Bunkyo-ku, Tokyo, 113-8510 Japan

**Keywords:** Senescence, Molecular biology, Cartilage

## Abstract

Osteoarthritis (OA) is an age-related degenerative joint disease that causes progressive cartilage loss. Chondrocyte senescence is a fundamental mechanism that contributes to the imbalance of matrix homeostasis in OA by inducing senescence-associated secretory phenotype (SASP). Although OA chondrocytes are mainly exposed to oxidative and inflammatory stresses, the role of these individual stresses in chondrocyte senescence remains unclear. In this study, we compared the effects of these stresses on the senescence of rat chondrocytes. Rat chondrocytes were treated with H_2_O_2_ and a combination of IL-1β and TNF-α (IL/TNF) to compare their in vitro effect on senescent phenotypes. For in vivo evaluation, H_2_O_2_ and IL/TNF were injected into rat knee joints for 4 weeks. The in vitro results showed that H_2_O_2_ treatment increased reactive oxygen species, γ-H2AX, and p21 levels, stopped cell proliferation, and decreased glycosaminoglycan (GAG)-producing ability. In contrast, IL/TNF increased the expression of p16 and SASP factors, resulting in increased GAG degradation. Intraarticular injections of H_2_O_2_ did not cause any changes in senescent markers; however, IL/TNF injections reduced safranin O staining and increased the proportion of p16- and SASP factor-positive chondrocytes. Our results indicate that oxidative and inflammatory stresses have significantly different effects on the senescence of rat chondrocytes.

## Introduction

Articular cartilage consists of chondrocytes and an abundant extracellular matrix (ECM), such as glycosaminoglycan (GAG) and type II collagen. Homeostasis in cartilage ECM is finely tuned by chondrocytes and their surrounding environments. The loss of homeostasis leads to the development of osteoarthritis (OA)^1^. OA is the most common joint disease in the world and is characterized by progressive cartilage loss and synovitis. Although its molecular mechanisms are not yet fully understood^2^, aging has been identified as one of the most important risk factors for the onset and progression of OA^3^.

Cellular senescence is a fundamental mechanism that contributes to the acceleration of aging-associated diseases, including OA. In OA cartilage, the number of senescent chondrocytes is correlated with disease severity^4^. The characteristics of senescent chondrocytes include morphological changes into flattened or amoeboid-like shape^5^, increased expression of senescence-associated β galactosidase (SA-β-gal)^6^, and accumulated DNA damage^7^. Senescent cells stop proliferating due to increased expression of cyclin-dependent kinases (CDKs), such as p16 and p21, upon various stresses^8^. An important characteristic of senescent cells is the increased release of inflammatory cytokines, proteases, and chemokines, which are collectively referred to as senescence-associated secretory phenotype (SASP) factors. In OA, SASP factors such as IL-6, MCP-1, and MMP-13 produced by senescent chondrocytes cause an imbalance between cartilage ECM synthesis and degradation, resulting in progressive deterioration of joint structure^9^.

Chondrocytes in OA joints are exposed to various senescence-inducing stresses. One of the main stresses is oxidative stress, which refers to the elevated level of reactive oxygen species (ROS), which is generated through aging and obesity^10^. ROS is involved in DNA damage, and its accumulation produces senescent chondrocytes^11^. Another main stress in OA is inflammatory stress. The OA synovium releases large amounts of various inflammatory cytokines^2^. Among them, IL-1β and TNF-α are particularly involved in the pathogenesis of OA^12^. IL-1β and TNF-α are reported to induce senescence in chondrocytes^13,14^. Thus, oxidative and inflammatory stresses are supposed to have a profound effect on chondrocyte senescence in OA. However, the roles of these individual stresses and their differences in the induction of chondrocyte senescence remain unclear. In this study, we compared the effects of oxidative and inflammatory stress on rat chondrocyte senescence, both in vitro and in vivo.

## Results

### Oxidative and inflammatory stresses increased the SA-β-gal activity in rat chondrocytes

**Figure 1:**
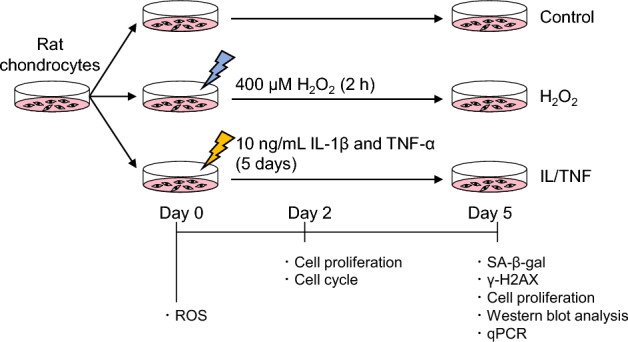
Experimental schema and timetable of the in vitro study. To induce oxidative stress, rat chondrocytes were treated 400 μM H_2_O_2_ for 2 h and then cultured in the growth medium without H_2_O_2_ (H_2_O_2_ group). To induce inflammatory stress, the cells were cultured in the growth medium supplemented with 10 ng/mL IL-1β and 10 ng/mL TNF-α (IL/TNF group). We performed reactive oxygen species (ROS) assay on day 0, cell proliferation and cell cycle assay on day 2, and senescence-associated β galactosidase (SA-β-gal) staining, γ-H2AX immunostaining, cell proliferation assay, western blot, and quantitative real-time PCR (qPCR) on day 5.

**Figure 2:**
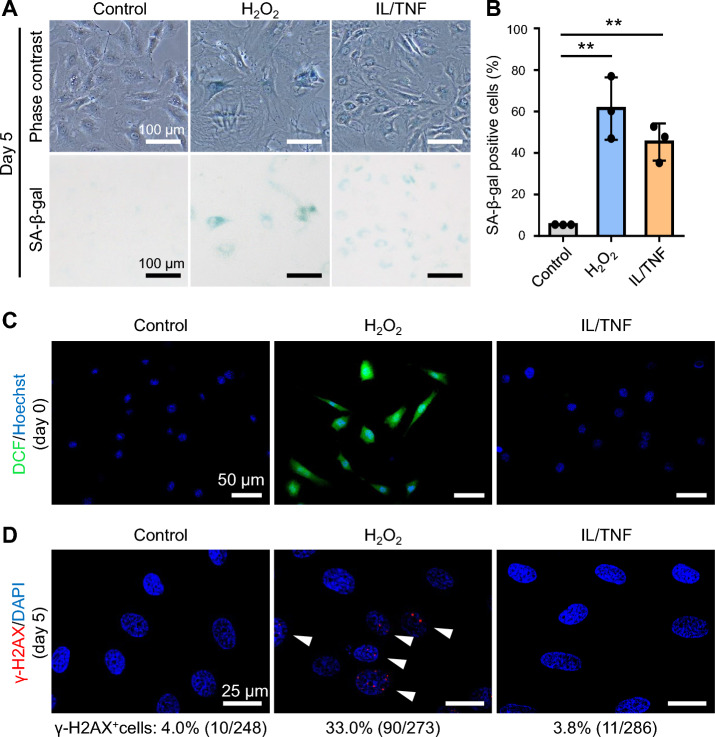
The level of senescence-associated β galactosidase (SA-β-gal), reactive oxygen species (ROS), and γ-H2AX. (**A**) Phase contrast and SA-β-gal (brightfield) images. (**B**) The proportion of SA-β-gal positive cells. Data are presented as mean ± SD of 3 independent experiments. **p < 0.01. (**C**) Detection of ROS by 2′,7′-dichlorodihydrofluorescein (DCF) fluorescence (green) with nuclei stained with Hoechst (blue). (**D**) γ-H2AX immunostaining (red). Nuclei were stained using DAPI (blue). White arrowheads indicate γ-H2AX positive cells.

An experimental scheme is shown in Fig. 1. We first assessed the SA-β-gal activity of rat chondrocytes after the induction of oxidative and inflammatory stresses. Cells became flat and large shaped in the H_2_O_2_ group and small and elongated shaped in the IL/TNF group compared to the control group (Fig. 2A). The ratio of SA-β-gal + cells in the H_2_O_2_ and IL/TNF groups was significantly higher than in the control group (Fig. 2B). The staining intensity was stronger in the H_2_O_2_ group than in the IL/TNF group.

### Oxidative stress increased the level of ROS and γ-H2AX

The ROS production in rat chondrocytes was evaluated using a DCFH-DA assay. No ROS were detected in either the control or IL/TNF groups, but treatment with H_2_O_2_ markedly increased the intracellular ROS level (Fig. 2C). We also assessed the expression of γ-H2AX which reflected the damage to DNA. Immunostaining showed that the proportion of γ-H2AX-positive cells was increased in H_2_O_2_-treated cells (Fig. 2D).

### Oxidative stress inhibited the proliferation of rat chondrocytes

**Figure 3:**
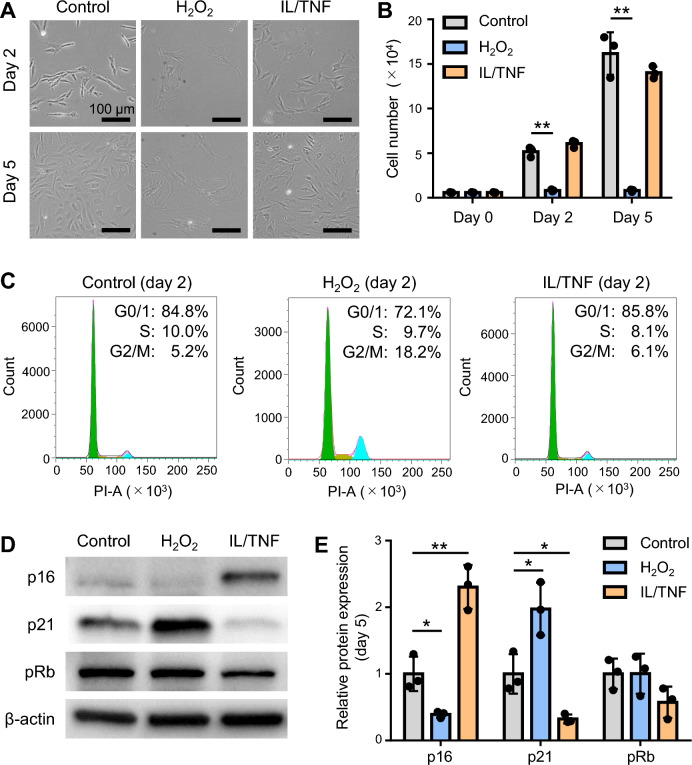
Cell proliferation and cell cycle. (**A**) Phase contrast images of cells on days 2 and 5. (**B**) The number of cells on days 0, 2, and 5. Data are presented as the mean ± SD of triplicate wells. **p < 0.01. (**C**) Cell cycle analysis using propidium iodide (PI). (**D**) Representative images of western blot for p16, p21, pRb, and β-actin. (**E**) Quantification of p16, p21, and pRb expression. β-actin was used as a loading control. Data are presented as mean ± SD of 3 independent experiments. *p < 0.05, **p < 0.01.

The effect of oxidative and inflammatory stresses on cell proliferation was examined via cell counting (Fig. 3A). No differences were observed in terms of cell number at days 2 and 5 between the control and IL/TNF groups (Fig. 3B). By contrast, H_2_O_2_-treated cells hardly proliferated, and the number of cells at days 2 and 5 were significantly lower in H_2_O_2_ group than in the control group. Cell cycle analysis using PI revealed that the proportion of cells in G2/M phase was higher in H_2_O_2_ group than in the control group (Fig. 3C). We evaluated the expression of cell cycle-related proteins by western blot analysis (Fig. 3D). The expression of p16 was significantly decreased in the H_2_O_2_ group and significantly increased in the IL/TNF group compared with the control group (Fig. 3E). Conversely, the expression of p21 was significantly increased in the H_2_O_2_ group and decreased in the IL/TNF group. There was no difference in the expression of pRb.

### Inflammatory stress increased the expression of SASP factors

**Figure 4:**
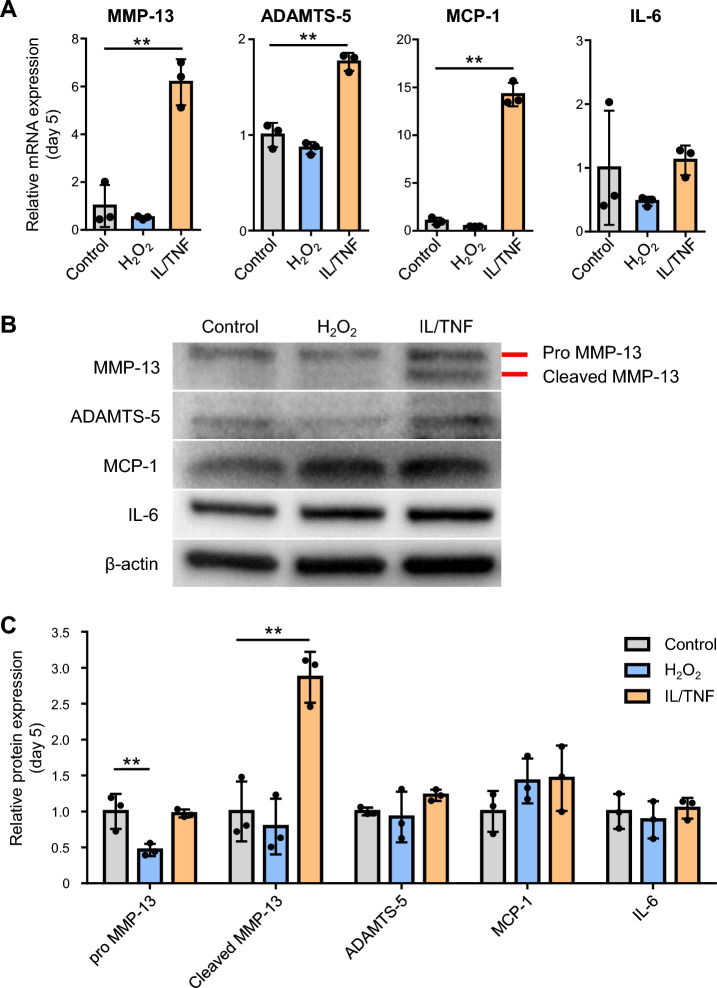
Expression of senescence-associated secretory phenotype (SASP) factors on day 5. (**A**) Quantification of mRNA expression of MMP-13, ADAMTS-5, MCP-1, and IL-6. Data are presented as the mean ± SD of triplicate dishes. **p < 0.01. (**B**) Representative images of western blot for MMP-13, ADAMTS-5, MCP-1, IL-6, and β-actin. (**C**) Quantification of MMP-13, ADAMTS-5, MCP-1, and IL-6 expression. β-actin was used as a loading control. Data are presented as mean ± SD of 3 independent experiments. **p < 0.01.

The mRNA expression of SASP factors (MMP-13, ADAMTS-5, MCP-1, and IL-6) was compared by qPCR. Inflammatory stress significantly enhanced the gene expression of MMP-13, ADAMTS-5, and MCP-1, but not of IL-6. No difference was observed in the expression of any of these genes between the H_2_O_2_ group and the control group (Fig. 4A). We examined the protein expression of SASP factors using western blots (Fig. 4B). The protein expression of cleaved MMP-13 was significantly higher in the IL/TNF group than in the control group, while that of the other genes was unchanged (Fig. 4C). Oxidative stress treatment did not affect the expression level of SASP factors except for decreasing pro MMP-13 expression.

### Oxidative stress impaired ECM-producing ability

**Figure 5:**
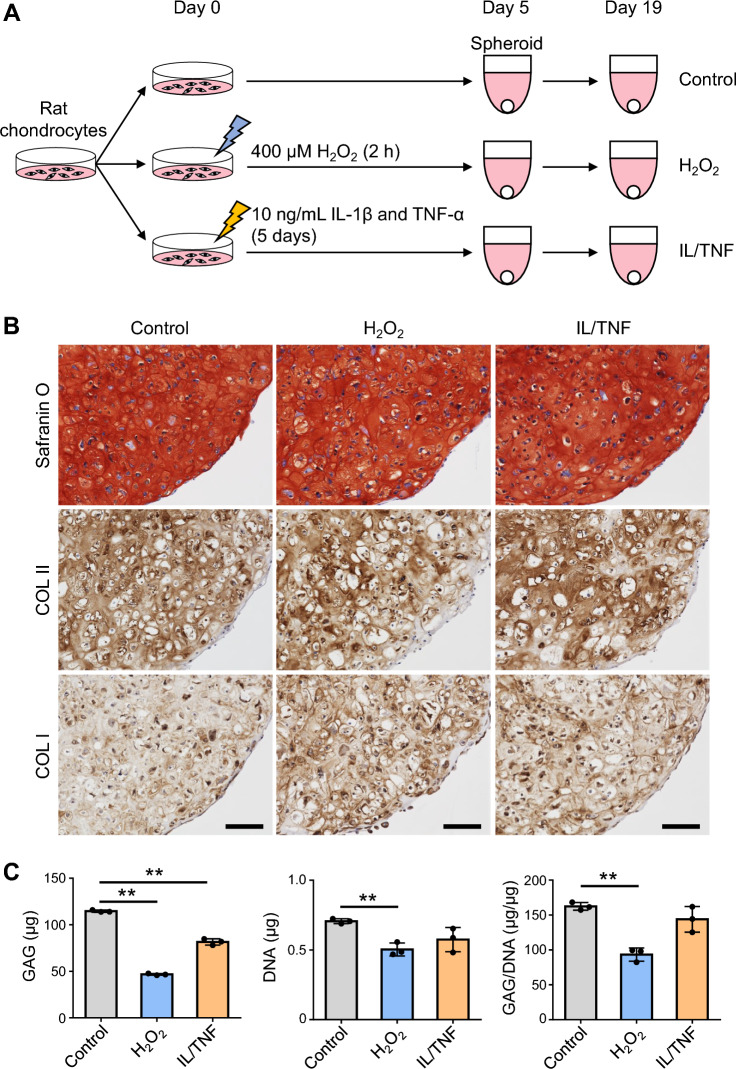
Production of the cartilage extracellular matrix. (**A**) Experimental schema. Rat chondrocytes were treated with H_2_O_2_ and IL-1β/TNF-α (IL/TNF); then, they were subjected to spheroid culture in a chondrogenic medium for 14 days. (**B**) Representative images of safranin O staining and immunostaining for type I and II collagen (COL I and II). (**C**) Quantification of glycosaminoglycan (GAG) and DNA. The GAG content was standardized to DNA content (GAG/DNA). Data are presented as the mean ± SD of triplicate spheroids. **p < 0.01.

We next generated chondrogenic spheroids from H_2_O_2_− and IL/TNF-treated chondrocytes to evaluate the effect of oxidative and inflammatory stresses on ECM-producing ability (Fig. 5A). Spheroids of all groups were strongly stained with safranin O (Fig. 5B). The intensity of type II collagen staining was equivalent in all groups. Greater type I collagen expression was observed in the H_2_O_2_ and IL/TNF group than in the control group, and spheroids from H_2_O_2_-treated cells showed significantly lower GAG and DNA amounts and GAG/DNA ratio than those in untreated cells (Fig. 5C). The GAG content was significantly lower in the IL/TNF group than in the control group; however, no difference was observed in the DNA content and GAG/DNA ratio.

### Inflammatory stress increased the degradation of ECM

**Figure 6:**
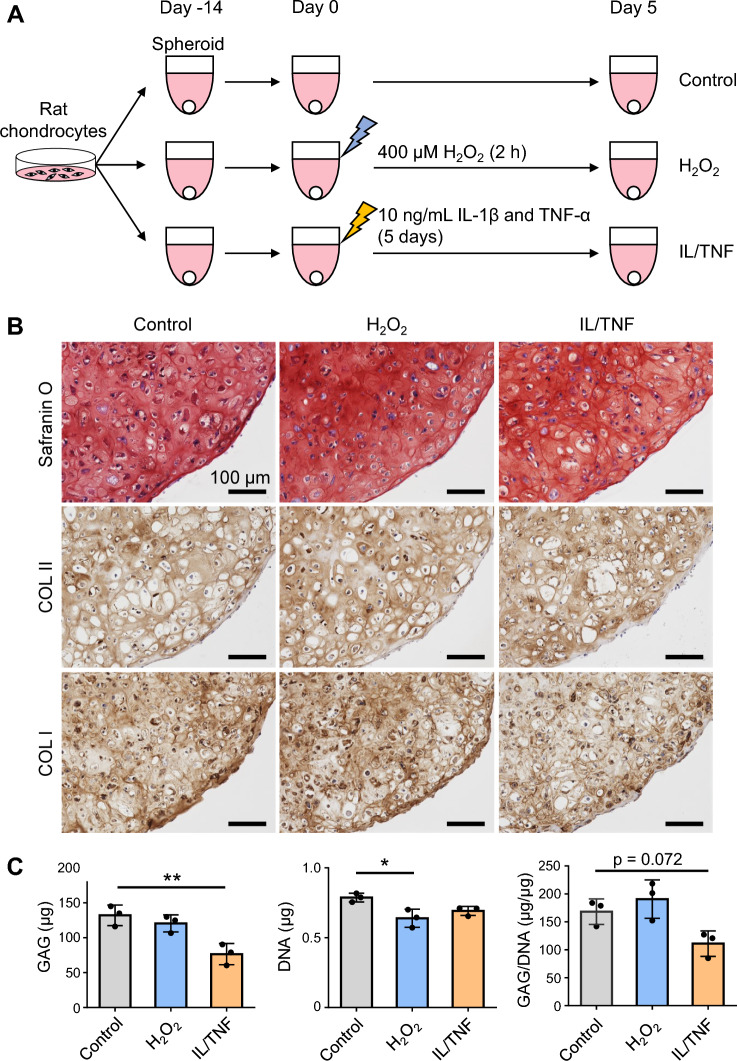
Degradation of the cartilage extracellular matrix. (**A**) Experimental schema. Chondrogenic spheroids were prepared from normal rat chondrocytes and then treated with H_2_O_2_ and IL-1β/TNF-α (IL/TNF). (**B**) Representative images of safranin O staining and immunostaining for type I and II collagen (COL I and II). (**C**) Quantification of glycosaminoglycan (GAG) and DNA. The GAG content was standardized to DNA content (GAG/DNA). Data are presented as the mean ± SD of triplicate spheroids. *p < 0.05, **p < 0.01.

The effect of oxidative and inflammatory stresses on cartilage ECM degradation was investigated by treating chondrogenic spheroids with H_2_O_2_ and IL/TNF (Fig. 6A). There were no obvious changes in the intensity of safranin O and type ll collagen staining among 3 groups (Fig. 6B). Spheroids treated with IL/TNF showed lower type I collagen expression than those in the control group. Furthermore, biochemical analysis revealed that the treatment of spheroids with IL/TNF significantly decreased the GAG amount (Fig. 6C). GAG/DNA ratios tended to be lower in IL/TNF group than in the control group. On the other hand, H_2_O_2_-treatment decreased the DNA amount but not the GAG amount and GAG/DNA ratio.

### Inflammatory stress increased the expression of p16 and SASP factors in vivo

**Figure 7:**
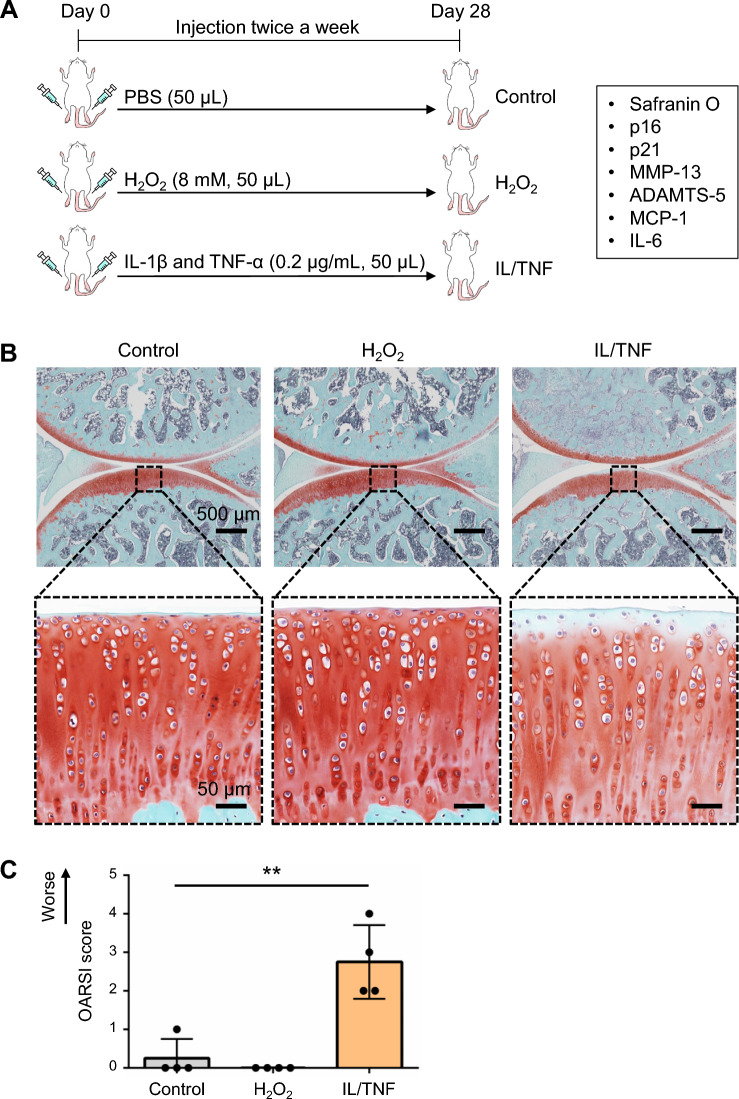
In vivo experimental schema and safranin O staining. (**A**) Experimental schema of the in vivo study. PBS, H_2_O_2_, and IL-1β/TNF-α (IL/TNF) were injected into the rats’ knee joints twice a week. Knee joint tissues were analyzed four weeks after the initial injection. (**B**) Representative images of safranin O staining of the knee joints (upper panels). The black dashed boxes represent enlarged images of tibial cartilage (lower panels). (**C**) OARSI score for tibial cartilage. Data are presented as the mean ± SD of 4 knees. **p < 0.01.

**Figure 8:**
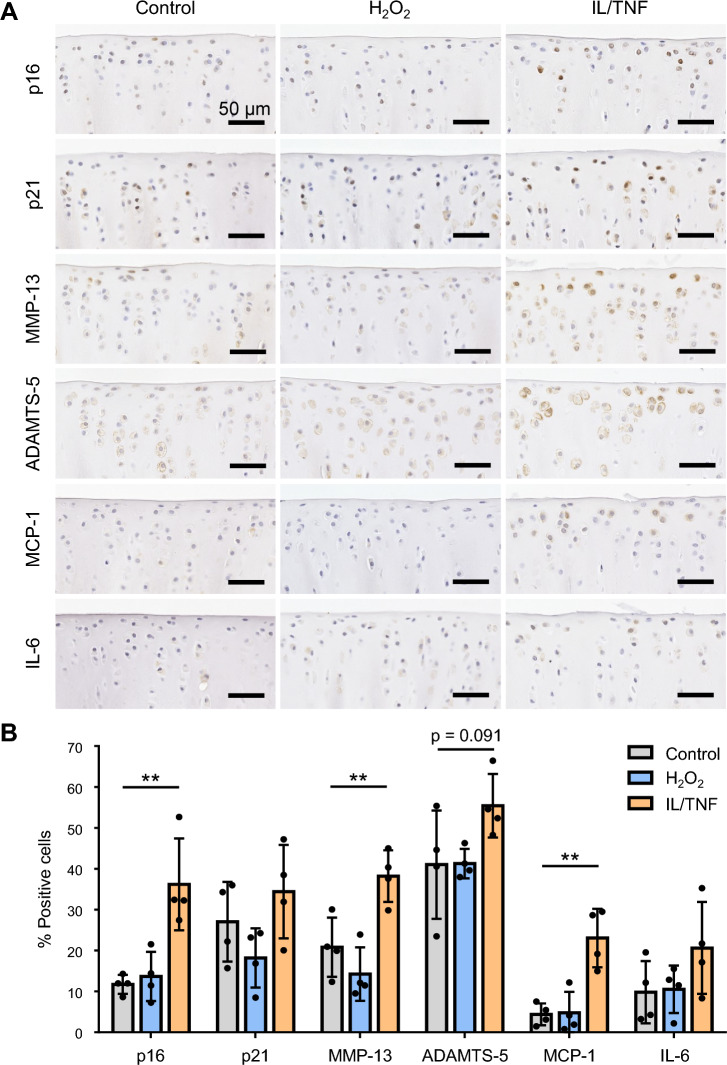
Immunostaining for senescence markers. (**A**) Representative images of p16, p21 MMP-13, ADAMTS-5, MCP-1, and IL-6 immunostaining. (**B**) Percentage of chondrocytes positive for p16, p21, MMP-13, ADAMTS-5, MCP-1, and IL-6. Data are presented as the mean ± SD of 4 knees. **p < 0.01.

H_2_O_2_ and IL/TNF were injected into rat knee joints to investigate the effects of oxidative and inflammatory stresses in vivo (Fig. 7A). In the control and H_2_O_2_ groups, the tibial articular cartilage was strongly stained with safranin O (Fig. 7B). However, IL/TNF injection reduced the staining of the cartilage, specifically on its surface. The Osteoarthritis Research Society International (OARSI) score was significantly higher in the IL/TNF group (Fig. 7C). The expression of senescence markers was also examined by immunostaining (Fig. 8A). The percentage of cells positive for p16, MMP-13, and MCP-1 was significantly higher in IL/TNF group than in the control group (Fig. 8B). ADAMTS-5 positive cells tended to be increased in IL/TNF group. H_2_O_2_ injection did not affect the expression of any evaluated markers.

## Discussion

Oxidative and inflammatory stresses increased the proportion of SA-β-gal positive cells in this study, indicating that both stresses induced the senescence of rat chondrocytes. The stronger staining intensity in the H_2_O_2_ group suggested that oxidative stress caused a greater expression of β-galactosidase than was induced by inflammatory stress. β-galactosidase is a lysosomal hydrolase that cleaves the terminal β-d-galactose residue, and its activity is upregulated in cells with senescent features accumulated in aged and diseased organs^15^. SA-β-gal activity is reported to increase in senescent chondrocytes^16^ and is now widely used as a biomarker for senescent cells across species and cell origins.

We found that oxidative stress increased the amount of intracellular ROS and expression of γ-H2AX which is present at DNA double-strand break sites^17^, while inflammatory stress did not. Elevated levels of ROS have been reported to impair the repair mechanism of damaged DNA in chondrocytes^18^. These findings suggest that oxidative stress induced DNA damage via ROS in rat chondrocytes, and inflammatory stress does not induce ROS production.

As shown in the proliferation and cell cycle assays, oxidative stress completely stopped cell division with G2/M arrest and increased p21 expression. Since ROS/p53/p21 pathway is well known to inhibit cell growth^19,20^, increased ROS levels in the present study would upregulate the expression of p21 to stop chondrocyte proliferation. Bunz et al. reported that increased p21 expression induced G2/M arrest in human colorectal cancer cell lines^21^, which is in line with our results. On the other hand, inflammatory stress increased p16 expression but did not alter the cell cycle or proliferation ability. IL-1β and TNF-α are known to induce p16 expression in rat chondrocytes and human chondrocyte cell lines, respectively^13,14^. Although p16 is generally considered to inhibit cell cycle progression^22,23^, Diekman et al. found that p16 expression was not associated with proliferation arrest in rat chondrocytes^24^, a result that is echoed in our findings. Thus, in rat chondrocytes, increased p16 levels do not always reflect decreased proliferative ability.

The release of SASP factors is one of the main characteristics of senescent cells. Real-time PCR analysis showed that inflammatory stress enhanced the mRNA expression of MMP-13, ADAMTS-5, and MCP-1. IL-1β and TNF-α have been reported to activate NFκB and MAPK and stimulate the production of these SASP factors^25–27^. On the other hand, western blot analysis showed that protein expression of MMP-13 was upregulated in the IL/TNF group but that of ADAMTS-5 and MCP-1 was not. As we used cell lysates in western blots, intracellular rather than extracellular protein levels were evaluated. ELISA might have been desirable for detecting SASP factors released outside the cells. In contrast to inflammatory stress, oxidative stress did not change the expression of any SASP factors we examined. The p53-p21 pathway is reported to suppress MAPK and NFκB signaling^28,29^, suggesting that oxidative stress alone cannot induce SASP in rat chondrocytes. Another possibility is that longer-term observation may be necessary after H_2_O_2_ treatment to confirm deep senescence.

Rat chondrocytes treated with H_2_O_2_ formed chondrogenic spheroids with a low GAG/DNA ratio. Decreased GAG production is one of the phenotypes observed in senescent chondrocytes^30^. Miki et al. demonstrated that ROS generated by mechanical stress suppressed proteoglycan synthesis in human chondrocytes^31^. We observed that IL/TNF treatment did not affect GAG-producing ability; instead, it promoted GAG degradation. IL-1β and TNF-α can induce the production of extracellular proteases such as MMP-13 and ADAMTS-5. It is suggested that these SASP factors are responsible for the degradation of GAGs in this study. Taken together, the effects of oxidative and inflammatory stress on GAG production and degradation differ from each other.

The injection of IL/TNF into rat knee joints decreased the intensity of safranin O staining in tibial cartilage, especially on the cartilage surface, and increased the OARSI score. In addition, the proportion of cells positive for p16, MMP-13, ADAMTS-5, and MCP-1 increased in the IL/TNF group. These data are consistent with in vitro results that inflammatory stress induced senescent phenotypes, such as the increased expression of p16 and SASP factors and GAG degradation. Increased p16 expression in chondrocytes has been reported to induce SASP^32^ but Diekman et al. recently found that p16 is just a biomarker, and not an inducer, for SASP in chondrocytes^24^. There is no consensus on the causal relationship between p16 and SASP factors.

In contrast, H_2_O_2_ injection did not affect the expression of any senescent markers, including p21, whose expression was upregulated in vitro. This indicates that oxidative stress alone cannot induce chondrocyte senescent phenotypes in vivo. We injected 8 mM H_2_O_2_, which was 20 times higher than the concentration used in vitro. Although a higher concentration of H_2_O_2_ may allow detection of changes in cartilage structures and senescent markers, it should have been noted that strong oxidative stress induces cell death instead of cellular senescence^33,34^. Another possibility is that injected H_2_O_2_ did not reach the chondrocytes. However, evaluation of the in vivo ROS distribution using tissue sections is difficult due to the rapid diffusion of ROS.

SASP is the most important senescence phenotype in OA progression because it triggers an imbalance in ECM production and degradation. Our findings suggest that inflammatory stress, rather than oxidative stress, is the dominant driver of SASP in OA. The selective elimination of senescent chondrocytes is a promising therapeutic approach for OA^35,36^. However, this approach targets the cells that already undergo senescence and therefore cannot prevent the cells from senescence itself. Regulation of inflammation stress, such as IL-1β and TNF-α signaling will be critical for inhibiting cellular senescence, particularly the acquisition of SASP.

In conclusion, oxidative stress induced DNA damage, increased p21 expression, arrested cell proliferation, and decreased GAG-producing ability in vitro, but caused no changes in senescent phenotypes in vivo. In contrast, inflammatory stress increased the expression of p16 and SASP factors and GAG degradation, both in vitro and in vivo. Our results demonstrated that the effects of oxidative and inflammatory stresses on the senescent phenotypes of rat chondrocytes differed significantly.

## Methods

### Rat chondrocyte isolation

All experiments and methods were performed in accordance with relevant guidelines and regulations. All animal care and experimental protocols were conducted in accordance with the ARRIVE guidelines and approved by the Animal Committee of Tokyo Medical and Dental University (approval number A2022-084A). Two 12-week-old male Lewis rats were obtained from the Sankyo Labo Service Corporation (Tokyo, Japan). Articular cartilage was harvested from knee joints and digested with 3 mg/mL *Clostridium histolyticum-*derived collagenase type V (C9263; Sigma-Aldrich, Saint Louis, MO, USA) at 37 °C for 2 h. The cells were filtered through a 70 μm cell strainer (542070; Greiner Bio-one GmbH, Kremsmuenster, Austria) and plated at a density of 5000 cells/cm^2^ in a growth medium consisting of Dulbecco’s Modified Eagles Medium (DMEM, D6046; Sigma-Aldrich), 10% fetal bovine serum (Lot no. 42F1562K; Thermo Fisher Scientific, Waltham, MA, USA), and 1% antibiotic–antimycotic (15240062; Thermo Fisher Scientific). After 10 days of cultivation, the cells were detached with 0.25% trypsin and 1 mM ethylenediaminetetraacetic acid (EDTA, 25200-072; Thermo Fisher Scientific) and cryopreserved with a cell banker, 1plus (CB021; Zenoaq, Fukushima, Japan), for future use.

### Induction of oxidative and inflammatory stress in chondrocytes

To induce oxidative stress, rat chondrocytes at passage 2 were treated with 400 μM H_2_O_2_ (080-01186; Wako, Tokyo, Japan) for 2 h and then cultured in the growth medium without H_2_O_2_ for 5 days (H_2_O_2_ group). To induce inflammatory stress, the cells were cultured in the growth medium supplemented with 10 ng/mL recombinant rat IL-1β (400-01B; PeproTech, Rocky Hill, NJ, USA) and TNF-α (400-14; PeproTech) for 5 days (IL/TNF group). The cells cultured in the growth medium were used as a control group.

### SA-β-gal staining

We performed SA-β-gal staining using a Senescence β-Galactosidase Staining Kit (#9860; Cell Signaling Technology, Danvers, MA, USA). Briefly, the cells were fixed with fixative solution for 10 min and incubated at 37 °C in staining solution at pH 6.0 for 16 h. SA-β-gal^+^ cells were counted in four fields at × 10 magnification with a microscope (BZ-X700, KEYENCE, Osaka, Japan).

### ROS detection

ROS levels were evaluated using a ROS Assay Kit (R252; Dojindo, Kumamoto, Japan). The cells were plated in an 8-well 0.8 cm^2^ Lab-Tek chamber slide (177445PK; Thermo Fisher Scientific). After 24 h, the cells were washed with phenol red-free Hanks’ balanced salt solution (HBSS, 084-08965; Wako) and incubated in DCFH-DA solution and 0.5 μg/mL Hoechst 33342 (H342; Dojindo, Tokyo, Japan) for 30 min at 37 °C with 5% CO_2_. Thereafter, the cells were washed with HBSS and treated with H_2_O_2_ and IL/TNF for 30 min. After washing with HBSS, the fluorescence signal was detected using a fluorescence microscope (BZ-X700, KEYENCE).

### Immunocytochemistry for γ-H2AX

Cells were fixed with 4% paraformaldehyde (158127; Sigma-Aldrich), permeabilized with 0.1% Triton X-100 (169-21105; Wako), and blocked with Blocking One Histo (06349-64; NAKALAI TESQUE, Kyoto, Japan) for 10 min. The cells were then incubated overnight with primary antibodies against γ-H2AX (1:200, #9718; Cell Signaling Technology) at 4 °C. After washing with phosphate-buffered saline (PBS) containing 0.1% Tween-20 (167-11515; Wako), goat anti-rabbit IgG (H&L) conjugated with Alexa Fluor 555 (1:200, ab150086; Abcam, Cambridge, UK) was applied for 1 h. Nuclei were stained with 1 μg/mL of 4′,6-diamidino-2-phenylindole (D523; Dojindo). The fluorescent signal was observed using a fluorescence microscope and γ-H2AX^+^ cells were counted manually in three fields at × 20 magnification.

### Cell proliferation assay

The cells were plated in a 6-well plate (353046; Corning, Glendale, AZ, USA) at a density of 1000 cells/cm^2^ in the growth medium. The next day (day 0), treatment with H_2_O_2_ and IL/TNF began. The number of viable cells was counted with an automatic cell counter (LUNA-FL; Logos, Gyeonggi-do, South Korea) at days 0, 2, and 5. Each experiment was performed in triplicate.

### Cell cycle assay

Cells on day 2 after the start of H_2_O_2_ and IL/TNF stimulation were fixed in ice-cold 80% ethanol (057-00456; Wako) for 30 min. The cells were then incubated overnight with 100 μg/mL RNase A (R6513; Sigma-Aldrich) and 50 μg/mL propidium iodide (PI, P3566; Thermo Fisher Scientific) at 4 °C. The fluorescence intensity of PI was evaluated by flow cytometry with a FACSVerse instrument (BD Biosciences, San Jose, CA, USA). The percentage of cells in G0/G1, S, and G2/M phases was calculated using FlowJo (BD).

### Western blot analysis

Cells were lysed in RIPA buffer (89900; Thermo Fisher Scientific) containing protease and phosphatase inhibitors (78440; Thermo Fisher Scientific). The protein concentration was calculated using a Pierce BCA Protein Assay Kit (23227; Thermo Fisher Scientific). 10 μg protein samples were loaded into Mini-PROTEAN TGX Precast Gels (#4569033; BIO-RAD) for electrophoresis. The separated proteins were transferred to Trans-Blot Turbo Mini PVDF membranes (#1704156; BIO-RAD). The membranes were then blocked with 5% bovine serum albumin (013-27054; Wako) or skim milk at room temperature for 1 h and incubated overnight with primary antibodies against p16 (1:1000, PA5-20379; Thermo Fisher Scientific), p21 (1:1000, ab80633; Abcam), pRb (1:1000, #8516; Cell Signaling Technology), MMP-13 (1:6000, ab39012; Abcam), ADAMTS-5 (1:1000, NBP2-15286; NOVUS Biologicals, Centennial, CO, USA), MCP-1 (1:1000, bs-1101R; Bioss Antibodies), IL-6 (1:1000, ab9324; Abcam), and β-actin (1:1000, #4970; Cell Signaling Technology) at 4 °C. After washing with Tris-buffered saline (TBS, #1706435; BIO-RAD) containing 0.05% Tween-20, secondary antibodies with horseradish peroxidase (1:5000, ab97040 and ab97080; Abcam) were applied for 1 h at room temperature. The membranes were then washed and applied with a SuperSignal West Femto Maximum Sensitivity Substrate (34095; Thermo Fisher Scientific). The ChemiDoc XRS + system (BIO-RAD) and Image Lab 6.1 (BIO-RAD) were used to detect chemiluminescent signals. The intensity of the chemiluminescence of each protein was normalized to that of β-actin. Original images of the immunoblots were shown in Supplementary Fig. S1.

### Quantitative real-time PCR (qPCR)

Total RNA was extracted using the miRNeasy Mini Kit (217004; QIAGEN, Venlo, Netherlands) according to the manufacturer’s instructions. Total RNA concentration was measured using NanoDrop (Thermo Fisher Scientific). First-strand complementary DNA was synthesized using a Transcriptor High Fidelity cDNA synthesis kit (5081955001; Roche Applied Sciences, IN, USA). The PCR was performed by real-time monitoring of THUNDERBIRD SYBR qPCR Mix (QPS-201; Toyobo, Osaka, Tokyo) on a LightCycler 480 instrument II (Roche Diagnostics, Mannheim, Germany). The following primers were used in this study: 18S ribosomal RNA, 5′-CACGGGTGACGGGGAATCAG-3′ (forward) and 5′-CGGGTCGGGAGTGGGTAATTTG-3′ (reverse); MMP-13, 5′-CTATGTCTGCCTTAGCTCCTGTC-3′ (forward) and 5′-CAACCCTGTTTACCTACCCACTTAT-3′ (reverse); ADAMTS-5, 5′-GCATCATCGGCTCAAAGCTACA-3′ (forward) and 5′-TCAGGGATCCTCACAACGTCAG-3′ (reverse); MCP-1, 5′-CAGCCAGATGCAGTTAATGCC-3′ (forward) and 5′-AGCCGACTCATTGGGATCAT-3′ (reverse); IL-6, 5′-TCCTACCCCAACTTCCAATGCTC-3′ (forward) and 5′-TTGGATGGTCTTGGTCCTTAGCC-3′ (reverse). The cycling conditions were 45 cycles at 95 °C for 15 s and 60 °C for 30 s. The 18S ribosomal RNA was used as an internal control.

### Chondrogenic spheroid culture

To evaluate the effect of oxidative and inflammatory stresses on the ECM-producing ability of rat chondrocytes, H_2_O_2_− and IL/TNF-treated cells were transferred into a prime surface 96-well plate (MS-9096U; Sumitomo Bakelite, Tokyo, Japan) at a density of 2.5 × 10^4^ cells/well to form spheroids and cultured in a chondrogenic medium consisting of high-glucose DMEM, 10 ng/mL transforming growth factor-β3 (130-094-008; Miltenyi Biotec, Bergisch Gladbach, Germany), 500 ng/mL bone morphogenetic protein-2 (7510050; Medtronic, MN, USA), 40 µg/mL proline (P0380; Sigma-Aldrich), 100 nM dexamethasone (047-18863; Wako), 100 µg/mL pyruvate (P5280; Sigma-Aldrich), 50 µg/mL ascorbate-2-phosphate (A8960; Wako), and 1% ITS Premix (354352; Becton, Dickinson and Company, NJ, USA). After 2 weeks of cultivation in chondrogenic medium, the spheroids were subjected to histological and biochemical analyses.

To evaluate the effect of oxidative and inflammatory stresses on cartilage ECM degradation, non-treated rat chondrocytes were transferred into a prime surface 96-well plate at a density of 2.5 × 10^4^ cells/well to form spheroids. They were then cultured in a chondrogenic medium for 2 weeks. Subsequently, chondrogenic spheroids were treated with H_2_O_2_ and IL/TNF. After 5 days from the start of the treatments, the spheroids were subjected to histological and biochemical analyses.

### DNA and GAG quantification

The spheroids were digested with 100 µg/mL papain (P3125; Sigma-Aldrich) at 65 °C for 16 h. DNA content was determined with Hoechst 33258 dye (H341; Dojindo). Fluorescence intensity was measured with a microplate reader (Infinite M200; Tecan, Männedorf, Switzerland) at an excitation wavelength of 360 nm and an emission wavelength of 465 nm. Calf thymus DNA (D4522; Sigma-Aldrich) was used to generate a standard curve. GAG content was determined using a Blyscan Kit (B1000; Biocolor, Westbury, NY, USA) according to the manufacturer’s instructions. The optical density at 656 nm was measured with a microplate reader. Finally, the total GAG content was also normalized to the total DNA content (GAG/DNA).

### H_2_O_2_ and IL/TNF injection into rat knee joints

Six male Lewis rats aged 11 weeks old were used. All rats were housed in cages containing two rats each. They were given food and water added libitum under a 12 h light/dark cycle, and acclimatized for 1 week. Rats were given H_2_O_2_ (8 mM, 50 μL) and IL/TNF (0.2 µg/mL, 50 µL) via an injection into their knees twice a week. These concentrations were set at 20 times the concentrations used in vitro. As a control, 50 μL of PBS was injected. After 4 weeks after the initial injection, the rats’ knee joints were analyzed histologically.

### Histology

Spheroids and knee joints were fixed in 10% neutral buffered formalin, embedded in paraffin, and sliced into 5-μm sections. The slides were stained with safranin O (1B463; Chroma Gesellschaft Schmid & Co., Munster, Germany) and fast green (061-00031; Wako) to visualize the distribution of GAG. Degeneration of the medial tibial cartilage was evaluated using the OARSI score^37^. For immunostaining, the slides were immersed in methanol (137-01823; Wako) containing 0.3% H_2_O_2_ to inhibit endogenous peroxidase activity. For p16, p21, MMP-13, ADAMTS-5, and IL-6, antigen retrieval was performed by immersing the sections in 10 mM Tris containing 1 mM EDTA (pH 9.0). For type I and II collagen, the sections were treated with 200 μg/mL proteinase K (161-28701; Wako) for 10 min. For type II collagen, an additional step was carried out: 5 mg/mL hyaluronidase (H3506; Sigma-Aldrich) was used for 1 h. Thereafter, the slides were blocked with Blocking One Histo (06349-64; Nacalai tesque, Kyoto, Japan) for 10 min and then incubated overnight with primary antibodies against p16 (1:200, ab54210; Abcam), p21 (1:200), MMP-13 (1:200), ADAMTS-5 (1:500), MCP-1 (1:200), IL-6 (1:200), type I collagen (1:200, 600-401-103-0.1; Rockland, Philadelphia, PA, USA), and type II collagen (1:200, F-57; Kyowa pharma chemical, Toyama, Japan) at 4 °C. After washing, the secondary antibodies with horseradish peroxidase (1:200, ab97040 and ab97080; Abcam) were applied for 1 h at room temperature. The sections were incubated with 3,3′-diaminobenzidine (K346811-2; Dako, Carpinteria, CA, USA), counterstained with hematoxylin (30002; Muto Pure Chemical, Tokyo, Japan), and observed with a microscope (BZ-X700). Cells positive for p16, p21, MMP-13, ADAMTS-5, MCP-1, and IL-6 in the medial tibial cartilage were counted at × 20 magnification.

### Statistical analysis

All statistical analysis was conducted using GraphPad Prism 8 software (GraphPad Software, CA, USA). One-way analysis of variance, followed by Dunnett's multiple-comparisons test, was used to compare the results for three groups. The P values < 0.05 were considered statistically significant.

## Supplementary Information

Supplementary Figure S1.

## Data Availability

All data generated or analyzed during this study are included in this published article.
