# Supplementary material for: Comparison of the effects of oxidative and inflammatory stresses on rat chondrocyte senescence
Source: Sci Rep. 2023 May 11;13:7697. doi: 10.1038/s41598-023-34825-1 (PMC10175275; doi:10.1038/s41598-023-34825-1)
Supplement: Supplementary file 1 — Supplementary Figure S1. [file 41598_2023_34825_MOESM1_ESM.pdf]

p16

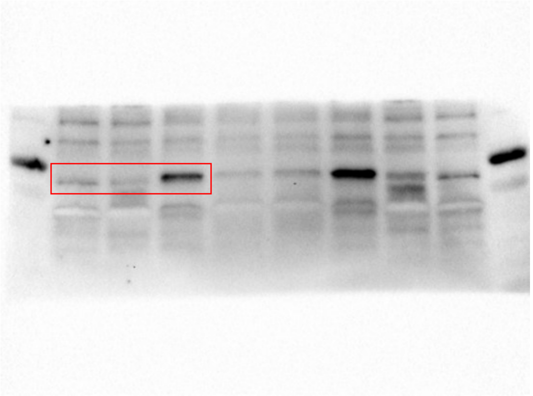

p21

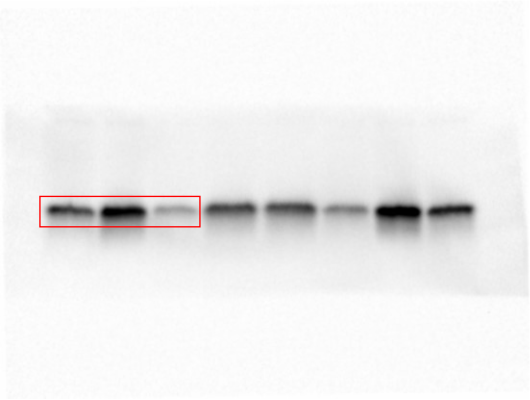

pRb

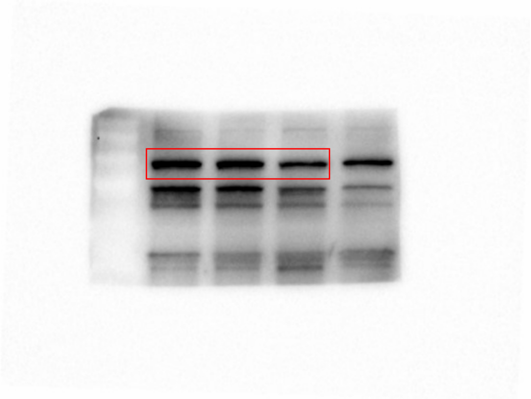

MMP-13

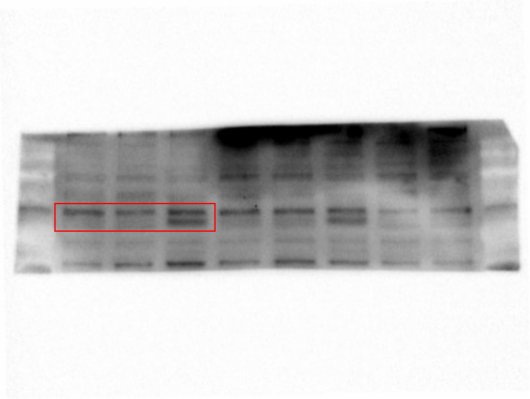

ADAMTS-5

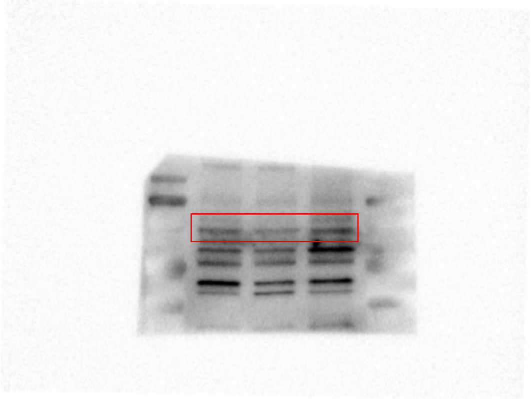

MCP-1

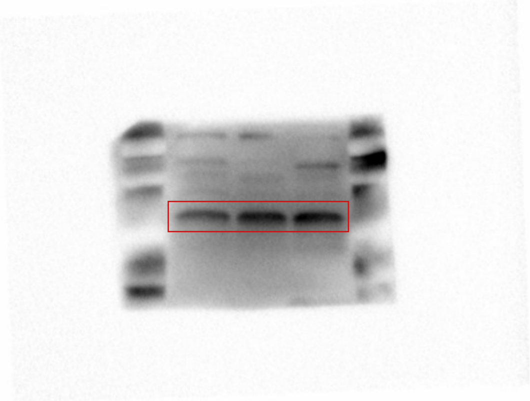

IL-6

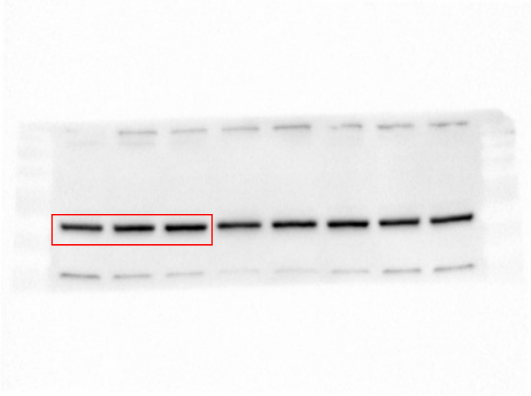

β-actin

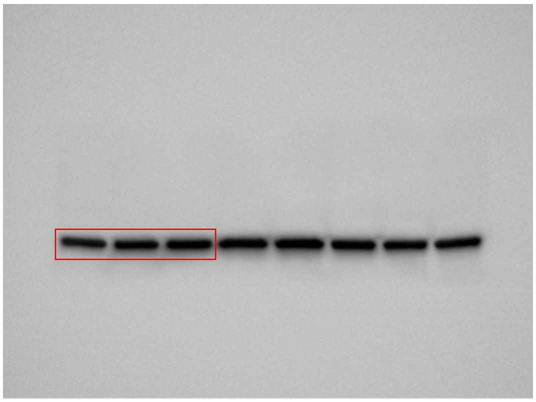

Supplementary Figure S1 | Original images of the immunoblots shown in Fig. 3D and 4B.
